# Supplementary material for: Y Chromosomes of 40% Chinese Descend from Three Neolithic Super-Grandfathers
Source: PLoS One. 2014 Aug 29;9(8):e105691. doi: 10.1371/journal.pone.0105691 (PMC4149484; doi:10.1371/journal.pone.0105691)
Supplement: Discussion S1 — Supporting Discussions. (DOCX) [file pone.0105691.s001.docx]

Additional Discussions:

###### *Precision in observing SNP occurrence*

Assuming the base substitution rate is constant, and each mutation is an independent random event, the observed occurrence of mutations from an ancestor to the descendant should follow Poisson distribution with expectation *λ*, which can be approximated as the observed mean of mutation occurrence. The standard deviation *σ* of Poisson distribution equals to$\sqrt{\lambda}$. When *λ* is a large number, the Poisson distribution can be simplified as a normal distribution $X\sim N(\lambda,\lambda)$. Thus the relative range of 95%CI is roughly $\pm\frac{2\sigma}{\lambda}=\pm\frac{2\sqrt{\lambda}}{\lambda}=\pm\frac{2}{\sqrt{\lambda}}$, i.e. in inverse proportion to the square root of expectation *λ*. We predict that when the whole NRY (~10 Mbp, with some regions prone to recurrent mutations) of these samples is sequenced in the future, the discovered SNPs would be roughly doubled, and the precision of date estimation would be enhanced by 1.4×, i.e. the 95%CI would be reduced from ±13.6% to less than ±10%.

###### *Estimation of captured range*

Considering both the positions on the designed bait regions and the 250 bp flanking regions by the baits, the range designed to be captured were 7.017 Mbp. Out of all the reads, 32 – 39% were mapped on this range (fluctuating among each pool). Since the size of whole human genome is about 3 Gbp and Y chromosome is haploid, the enrichment efficiency was about 300 times.

###### *Estimation of false positive and false negative rates of newly discovered SNPs*

We tested 36 newly discovered SNPs under Haplogroup N or the 002611+ clade, all of which were shared by more than one tested samples and conform to the phylogenetic tree. All these SNPs were validated by Sanger sequencing, and are thus highly reliable. We also tested 19 new private SNPs (found derivative in only one sample with at least 4× coverage), all but one were validated by Sanger sequencing. The false private SNPs could be due to any of sequencing error, mismatch during PCR steps, mapping error to homologous sequence, or misalignment near indels.

False negatives, i.e., undiscovered SNPs inside the sequenced 3.9 Mbp region can also be a factor that affects divergence time calculation. Specifically, most of those false negatives may exist in the terminal branches, or the private SNPs, due to low sequencing depth. Our procedure should have lowered this effect. Firstly, only the 47 individuals with averagely >6× depth and without clear traces of barcode confusion (in bold italic in Fig. 1 and Fig. S1) were used for time estimation, and other 31 sequences with moderate quality (less depth or with slight traces of barcode confusion) were kept in the tree in delivering more phylogenetic information but not used for time estimation. Due to the fact that Y chromosome is haploid, for the non-repeated regions of Y chromosome, under a condition of an error rate of 0.1% (Illumina), 2× sequencing depth should be enough to discover a new mutation, and 1× is enough for genotype calling. Therefore the criterion we used (on average >6× after removing duplicates) should not cause many false negatives. Secondly, the individual with best sequencing quality, YCH53, with 239× depth sequenced in a separate lane, did not show significantly longer branch than other individuals, indicating that 6× depth should be enough to avoid false negatives. In fact, most problems of false negatives were attributed to intermingling between samples due to the sequencing quality of barcodes, and thus private SNPs showed heterozygous and could sometimes not be effectively discovered. However, those individuals with any traces of intermingling were excluded from time estimations.

###### *Bottleneck and expansion viewed from the tree*

The time and shape of divergences in the phylogenetic tree along with the information of extant geographic distribution may provide information on when, where and how the haplogroups resided and developed. Whether an ancient expansion would be observed today depends not only on how strong the expansion was, but also on how the descendants survived in the later periods. Since the expansion of a population is an intrinsic continuous property, the final outcome of an expansion depends on the environment. A line segment on the tree without divergence during a long time may indicate a strong bottleneck occurred at the later end of the segment. On the other hand, a strong expansion during a short time does not simply mean that the period is extraordinary suitable for this clade, but rather that since this expansion, no prevalent and vital bottleneck has happened to counteract this expansion. Therefore, the stable food supply that was profited from agriculture likely provided the prerequisite of the successful star-like expansions. The time and shape of a divergence event may indicate whether the environment befits the survival. For example, frequent splits on a certain clade during the LGM period may indicate a southern (warmer) location, while an expansion that only happened several thousand years later than the LGM may indicate a relatively northern existence.

###### *A refined history of the haplogroups*

According to the divergence times calculated from the tree (Fig. S1) and the present geographical distribution of each haplogroup, a renewed migration route of East Eurasian haplogroups was proposed (Fig. S2 a – d).

It remained mysterious that how many times the anatomically modern human migrated out of Africa, since that among the three super-haplogroups C, DE and F, Haplogroup F distributes in whole Eurasia, C in Asia and Austronesia, D exclusively in Asia, while D’s brother clade E distribute mainly in Africa [63], so there are two hypotheses, 1) haplogroups D and CF migrated out of Africa separately; 2) the single common ancestor of CF and DE migrated out of Africa followed by a back-migration of E to Africa. From this study, the short interval between CF/DE and C/F divergences weakens the possibility of multiple independent migrations (CF, D, and DE*) out of Africa, and thus supports the latter hypothesis [64] (Fig. S2 a).

Haplogroup D is comprised of subclade D1-M15 and D3-P99, both in continental East Asian, especially frequent in Tibet [64], subclade D2-M55, nearly exclusively in Japan, and paragroup D*, which was discovered mainly in Tibet as well as on the Andaman Islands. In this study, only D1 and D3 samples were sequenced. The divergence time of D1 and D3 was at ~36 kya. Except for the sample YCH177 (Zhuang ethnicity), all the tested D1 samples (Han and Yi ethnicities) are derivative at SNP N1 [65].

Together with Haplogroup D, C is also considered as one of the harbingers in East Eurasia and Australia [66]. Soon after its divergence with F, Haplogroup C moved eastwards along the coast of Indian Ocean, reached India and China, and might be associated with the earliest known modern human inhabitants in Australia at 46 kya [67]. In China, the vast majority of Haplogroup C belongs to C3-M217 [68], which constitutes ~10% of Han Chinese, as well as great part of Altaic-speaking populations, e.g. Mongol, Manchu, and Kazakh. Here we identified two clades of C3 which split at 25.9 kya: a northern clade (C3-n) with SNP F1396, including a Mongol and a Manchu sample, and a southern clade (C3-s) with SNP F1144, including all sequenced Han Chinese C3 samples. The STRs of YCH168 (Mongol ethnicity) is close to the ‘star-cluster’, which is abundant in the steppe ethnicities [69], indicating that a substantial part of Altaic-speaking population belongs to C3-n. The southern clade expands rather late (only about 6.5 kya, i.e. in the Neolithic Age), including most former C3* individuals in Han Chinese. Interestingly, the subclade C3d-M407, which is common in Sojot (Turkic) and Buryat (Mongolic)[70], originated only after this expansion of C3-s. The C3-s clade showed a similar expansion time comparing to the three star-like expansions under O3, and probably will also be found a multifurcation, if more samples will be sequenced.

The Superclade F did not undergo major split since 54.0 kya, until the divergence of Haplogroup G and IJK at 35.8 kya, which was followed by the emergence of all major haplogroups (IJ, and K, and its subclades NO, P, and LT) during the following 3,000 years. Considering the present distributions of these haplogroups, we suggest that the expansion occurred in Central or South Asia. Since 30 kya, the Eurasians became adapted to colder environment and thrived in more northern areas like Europe and North China (Fig. S2 b), despite of the coming last glacial maximum (LGM, around 20 kya). Haplogroup NO migrated to East Asia and split into N and O at 30.0 kya.

Haplogroup P diverged into Q and R at ~24.1 kya, slightly before the LGM. Most Q individuals in Han Chinese belong to the Q1a1-M120 clade, while R’s in Han Chinese are mostly R1a1-M17. The separation events of R1 and R2, and R1a and R1b are estimated here at 19.9 and 14.8 kya, respectively. R1b roamed till the Atlantic coast, forming some of the non-Indo-European groups (e.g. Basque)[32].

This study leads to a discovery of 265 new SNPs under Haplogroup N-M231, adding significantly to the only 11 currently known SNPs [13]. Haplogroup N is frequently found in Tibeto-Burman [71,72], Austroasiatic [73], Altaic [74], Uralic [75], Slavic [76,77], and Baltic peoples [78,79]. Haplogroup N went through a bottleneck lasting for 14 thousand years (30 – 15.8 kya). Considering the last glacial period at 26.5 – 19 kya, when living space for human was probably very limited in the northern part of Asia, we therefore assume that at the LGM, the Haplogroup N survived in an area northern to Haplogroup O, e.g. the northern part of China. The expansion of Haplogroup N occurred at 16 – 13 kya, during which N1c-M46 and N1a-M128 separated. The former defining SNP of Haplogroup N1, LLY22g (double copied, CC > CA) is proved to be prone to recurrent mutation, since the sample YCH142, downstream of F842 clade, has CC genotype at LLY22g, while the other sequenced N samples have CA. The AA genotype was also observed in an N sample (genotyped in this study but not sequenced by next-generation method). It seems that CC and AA genotypes under Haplogroup N were results of homologous recombination of the two copies. Therefore, we suggest to remove the SNP LLY22g from the current phylogenetic tree as the case of SNP P25, which has 3 copies on Y chromosome [80], and the phylogeny of Haplogroup N is to be rearranged using the single copied SNPs discovered in this study.

Haplogroup O, which covered 1/4 of all males on the world today [20], began frequent splitting into subclades before the LGM. The ancestor of O-M175 suffered an intermediate bottleneck event at 30 – 25 kya, and expanded rapidly at 24.7 – 21.5 kya, indicating a southern distribution during the LGM. We found that Haplogroups O1 and O2 share 6 SNPs (e.g. F75), forming a monophyletic lineage before joined with O3-M122 (the current nomenclature is not changed in this study). O1a1-P203 is the major clade of Haplogroup O1 in China, especially frequent (>20%) in the eastern provinces like Zhejiang and Jiangsu [20], corresponding to the Neolithic expansion of the ancient Yue (ancestral group of present Tai-Kadai and southern Han Chinese) [81]. The major expansion under P203 in Han Chinese population (F78 clade) occurred at about 5 kya, younger than the three star-like expansions in O3, fitting to the rice-growers in Liangzhu culture (5.3 – 4.0 kya). Since Tai-Kadai O1 samples were not included in this study, their divergence time with the East China Yue population is not yet clear.

Among the three main branches of Haplogroup O, O2 clade expanded the earliest, fitting the current distribution which is more at the south. All the sequenced O2-M268 samples other than O2b-M176 form a monophyletic clade, labeled by F1462, and the SNP PK4 lies inside this clade. Further genotyping of the newly discovered SNPs under F1462 clade will unveil the origin and migration routes and time of the Austro-Asiatic and Tai-Kadai peoples in South China, Southeast Asia and India [82].

Haplogroup O3 covers more than half of all the Han Chinese population [20]. Except for a few O3*-M122(xM324) individuals (sharing the SNP F742), all the sequenced O3 samples are found belonging to any of the three clades KL1+, M188+, or P164+, which diverged at ~18 kya. The SNP M188, which was defined as downstream of M7 [13], is now found upstream of M7. Although no M159-derived samples were sequenced in this study, it has been revealed that M188 is also upstream of M159 [83]. Therefore, M7 and M159 should be parallel and under M188, which is fraternal to P164 and under P201. Under the P164 clade, N6 [65] defines a monophyletic clade parallel to M134. It would be interesting to know the detailed subclade of the Austronesian P201*(xM134,M7) clade [23,84], in order to know their relationship to the continental peoples. All the sequenced M134xM117 samples also showed a monophyletic group with derived allele at F444. There are also secondary bifurcations under L127 and IMS-JST002611 (in short, 002611).

Supplementary References:

63. Underhill PA, Kivisild T (2007) Use of Y chromosome and mitochondrial DNA population structure in tracing human migrations. Annu Rev Genet 41: 539-564.

64. Shi H, Zhong H, Peng Y, Dong YL, Qi XB, et al. (2008) Y chromosome evidence of earliest modern human settlement in East Asia and multiple origins of Tibetan and Japanese populations. BMC Biol 6: 45.

65. Deng W, Shi BC, He XL, Zhang ZH, Xu J, et al. (2004) Evolution and migration history of the Chinese population inferred from Chinese Y-chromosome evidence. J Hum Genet 49: 339-348.

66. Hudjashov G, Kivisild T, Underhill PA, Endicott P, Sanchez JJ, et al. (2007) Revealing the prehistoric settlement of Australia by Y chromosome and mtDNA analysis. Proc Natl Acad Sci USA 104: 8726-8730.

67. Bowler JM, Johnston H, Olley JM, Prescott JR, Roberts RG, et al. (2003) New ages for human occupation and climatic change at Lake Mungo, Australia. Nature 421: 837-840.

68. Zhong H, Shi H, Qi XB, Xiao CJ, Jin L, et al. (2010) Global distribution of Y-chromosome haplogroup C reveals the prehistoric migration routes of African exodus and early settlement in East Asia. J Hum Genet 55: 428-435.

69. Zerjal T, Xue YL, Bertorelle G, Wells RS, Bao WD, et al. (2003) The genetic legacy of the Mongols. Am J Hum Genet 72: 717-721.

70. Malyarchuk B, Derenko M, Denisova G, Wozniak M, Grzybowski T, et al. (2010) Phylogeography of the Y-chromosome haplogroup C in northern Eurasia. Ann Hum Genet 74: 539-546.

71. Hammer MF, Karafet TM, Park H, Omoto K, Harihara S, et al. (2006) Dual origins of the Japanese: common ground for hunter-gatherer and farmer Y chromosomes. J Hum Genet 51: 47-58.

72. Sengupta S, Zhivotovsky LA, King R, Mehdi SQ, Edmonds CA, et al. (2006) Polarity and temporality of high-resolution Y-chromosome distributions in India identify both indigenous and exogenous expansions and reveal minor genetic influence of central Asian pastoralists. Am J Hum Genet 78: 202-221.

73. Zhong H, Shi H, Qi XB, Duan ZY, Tan PP, et al. (2011) Extended Y chromosome investigation suggests postglacial migrations of modern humans into East Asia via the northern route. Mol Biol Evol 28: 717-727.

74. Pakendorf B, Novgorodov IN, Osakovskij VL, Danilova AP, Protod'jakonov AP, et al. (2006) Investigating the effects of prehistoric migrations in Siberia: genetic variation and the origins of Yakuts. Hum Genet 120: 334-353.

75. Wells RS, Yuldasheva N, Ruzibakiev R, Underhill PA, Evseeva I, et al. (2001) The Eurasian Heartland: A continental perspective on Y-chromosome diversity. Proc Natl Acad Sci USA 98: 10244-10249.

76. Balanovsky O, Rootsi S, Pshenichnov A, Kivisild T, Churnosov M, et al. (2008) Two sources of the Russian patrilineal heritage in their Eurasian context. Am J Hum Genet 82: 236-250.

77. Mirabal S, Regueiro M, Cadenas AM, Cavalli-Sforza LL, Underhill PA, et al. (2009) Y-Chromosome distribution within the geo-linguistic landscape of northwestern Russia. Eur J Hum Genet 17: 1260-1273.

78. Rootsi S, Zhivotovsky LA, Baldovič M, Kayser M, Kutuev IA, et al. (2007) A counter-clockwise northern route of the Y-chromosome haplogroup N from Southeast Asia towards Europe. Eur J Hum Genet 15: 204-211.

79. Tambets K, Rootsi S, Kivisild T, Help H, Serk P, et al. (2004) The western and eastern roots of the Saami - The story of genetic "outliers" told by mitochondrial DNA and Y chromosomes. Am J Hum Genet 74: 661-682.

80. Adams SM, King TE, Bosch E, Jobling MA (2006) The case of the unreliable SNP: Recurrent back-mutation of Y-chromosomal marker P25 through gene conversion. Forensic Sci Int 159: 14-20.

81. Li H, Wen B, Chen S-J, Su B, Pramoonjago P, et al. (2008) Paternal genetic affinity between western Austronesians and Daic populations. BMC Evol Biol 8: 146.

82. Kumar V, Reddy ANS, Babu JP, Rao TN, Langstieh BT, et al. (2007) Y-chromosome evidence suggests a common paternal heritage of Austro-Asiatic populations. BMC Evol Biol 7.

83. Rozen S, Marszalek JD, Alagappan RK, Skaletsky H, Page DC (2009) Remarkably Little Variation in Proteins Encoded by the Y Chromosome's Single-Copy Genes, Implying Effective Purifying Selection. Am J Hum Genet 85: 923-928.

84. Loo JH, Trejaut JA, Yen JC, Chen ZS, Lee CL, et al. (2011) Genetic affinities between the Yami tribe people of Orchid Island and the Philippine Islanders of the Batanes archipelago. BMC Genet 12: 21.
